# Implementation of a stroke/TIA e-consult increases outpatient stroke Teleneurology consults placed in VHA compared to community care

**DOI:** 10.3389/frhs.2026.1839089

**Published:** 2026-07-22

**Authors:** Linda S. Williams, Laura J. Myers, Joanne Daggy, Qing Tang, Jessica Kirchgassner, Grace Bastin, Fadzai Chagwena, Stan Taylor, Karen Odrzywolski, Lisa Nobel, Teresa M. Damush, Jayne R. Wilkinson

**Affiliations:** 1 VA HSR EXTEND QUERI, Indianapolis, IN, United States; 2 Department of Neurology, Indiana University School of Medicine, Indianapolis, IN, United States; 3 Regenstrief Institute, Inc, Indianapolis, IN, United States; 4 VA National TeleNeurology Program, Philadelphia, PA, United States; 5 Department of Neurology, University of Rochester School of Medicine, Rochester, NY, United States; 6 Corporal Micheal J. Crescenz VA Medical Center, Philadelphia, PA, United States; 7 Department of Neurology, University of Pennsylvania School of Medicine, Philadelphia, PA, United States

**Keywords:** cerebrovascular disease, electronic consultation, implementation, neurology, telehealth

## Abstract

**Background:**

E-consults for outpatient stroke/TIA care can improve timeliness of neurologic care. For rural Veterans, many of whom are eligible for VA-paid community care, it is unknown whether active implementation of an e-consult within the Veterans Health Affairs (VA) heath system impacts the overall volume of outpatient stroke care provided in the VA vs. in non-VA community care.

**Methods:**

We studied the implementation of an e-consult for outpatient stroke/TIA care in a stepped-wedge trial in 10 VA facilities in the VA National TeleNeurology Program (NTNP). Sites were randomized to one of three sequential six-month active implementation waves (after two months of usual care where the e-consult was available) that included a stroke prevention lecture for primary care (PC) providers, as well as participation in monthly PC meetings to review cases and utilization of the consult. After the six-month active implementation (sustainability period), no data were presented to PC teams. The primary outcome was whether a consult was placed in the VA vs. community care neurology (CCN) for outpatient stroke/TIA care. A generalized linear mixed model (GLMM) with binomial distribution and log link fit to the primary outcome was used to estimate the effect of active implementation with baseline serving as the reference. Other effects in the model included *a priori* selected variables: demographics, site data (stroke volume, median consult wait time, rurality, availability of local neurology, indicator for project time period—baseline, active, sustainability), time block (as categorical two-month blocks) and random site effect. A similar model was fit including four control sites, with an added site-level indicator for intervention vs. control site.

**Results:**

Of the 1,680 consults placed for stroke/TIA, 22% (365/1,680) were NTNP (within VA). The GLMM model demonstrated that the odds of a stroke/TIA consult being placed within the VA increased during active implementation: OR 1.92 (1.17–3.16, *p* = 0.01) but this effect did not remain significant during sustainability (OR 1.85, 95% CI 0.83–4.13). Adding data from the four NTNP control sites who had access to the e-consult but did not receive the active implementation showed no trend in consult location over time but added additional variability to the model, resulting in a decrease in the effect (OR 1.48, 95% CI 0.96–2.28, *p* = 0.07).

**Conclusion:**

Active implementation of a VA stroke/TIA e-consult may increase the proportion of neurology consults placed to VA compared to community care. Further implementation efforts to sustain this effect and better understand factors influencing patient and provider choices about where to receive care are needed.

## Introduction

Multiple studies have reported that neurologists are increasingly scarce and that access to neurologic care is becoming more difficult (1–4). Telehealth care, and specifically electronic consultation (e-consult), is among the approaches to addressing this shortage, as it can make care more timely and can overcome geographic and physical barriers to traveling for care (5, 6). The clinical effectiveness of telehealth care is reported to be similar across many disciplines compared to usual in-person care, although the effect of telehealth care availability on patterns of referral and utilization is less certain (7, 8). Teleneurology care is used for general and subspecialty outpatient neurology care, with use accelerating during the COVID-19 pandemic (9, 10). The VA implemented the National Teleneurology Program (NTNP) in 2019 to provide general neurology outpatient video telehealth care for Veterans at primarily rural and underserved VA facilities, demonstrating successful implementation, high acceptance rates among Veterans, and improvements in neurology access among participating facilities (11–13). NTNP is currently active in 15 sites across the United States, and in Fiscal Year (FY) 2025 conducted over 10,000 total outpatient neurology visits with almost 3,000 new patient visits.

As an addition to the ongoing Teleneurology video consults in FY2021, NTNP developed an e-consult for stroke/TIA care. E-consults are asynchronous written communications between providers with the request for consultation and consult documentation typically occurring within a shared health record system; e-consults may be implemented to attempt to improve specialty care access (6). We identified stroke as a good clinical condition for outpatient neurology e-consultation in NTNP since it is a common condition with risk factors typically managed by Primary Care providers (PCPs). Additionally, a prior study had also shown that patients with stroke may be less likely to receive neurology care compared to patients with other neurologic conditions (2) so this e-consult sought to address that potential gap for rural Veterans.

Veterans, especially those residing in rural areas, increasingly receive VA-paid healthcare outside the VHA system. Most recently, the 2018 Mission Act provided additional funding and organization for VA paid non-VA (community) healthcare networks (14). Eligible Veterans include those who live at a significant distance and/or drive time from the specialty care provider, where there is no nearby VA specialty care provider, or where wait time for a VA specialty provider is prolonged. When VA and non-VA care have been compared on various outcomes and conditions, most studies have found similar or higher care quality in VA compared to non-VA settings (15–17), but what factors drive care choices for Veterans and providers are less systematically studied. One review of studies that have examined patient and provider experiences related to expanding access to VA-paid community care found perceived benefits in access and choice, but also challenges in scheduling, care coordination and communication (18). A specific healthcare provider concern identified in some studies was the additional workload needed to review records and coordinate care across healthcare systems (19, 20). This observation suggests that VA primary care providers may view additional types of within-VA consultations, like e-consults, as especially useful when faced with a patient needing specialty care referral. In this project, we sought to determine whether the implementation of the new e-consult was associated with an overall increase in neurology consults for outpatient stroke/TIA care being placed to NTNP. Our hypothesis was that increasing early access to VA neurology care through an e-consult might increase the overall proportion of stroke/TIA consults that were placed to NTNP vs. those placed to community care neurology (CCN, non-VA care).

## Methods

This project was conducted as a quality improvement activity as part of the implementation and evaluation of the VA National TeleNeurology Program (NTNP) and the EXTEND QUERI program. The EXTEND QUERI program was a five-year VA Health Systems funded program focused on improving Veterans' access to care by implementing evidence-based practices via telehealth interventions in VA specialty care settings. The program included three projects; the stroke/TIA e-consult project described here was one of these. The project was determined to be a non-research activity by VHA QUERI and local IRB officials (Indiana University IRB Protocol #28362). We used the extension to the 2010 CONSORT statement for stepped wedge cluster randomized trials as a guide to study design and documentation (21).

The NTNP is a clinical program in VA funded by the VA Office of Rural Health and designed as a central hub with general neurologists conducting video telehealth visits, either directly to the Veteran's home or to rural clinics, for Veterans in need of neurologic care. Many Veterans at participating facilities also qualify for CCN referral, based on wait times and/or drive times to the nearest VA with neurology services, so Veterans may be eligible to choose that option for their outpatient neurology care.

### E-consult development

In 2021 NTNP developed a routine outpatient stroke/TIA e-consult for PCPs at all active facilities. We worked with VA Clinical Applications Coordinators and PCP advisors to ensure the consult was clear, promoted communication about the primary question(s) the PCP wanted the neurologist to address, and was placed in the appropriate location in each facility's electronic medical record. The consult included a clear reminder that urgent stroke/TIA questions were not appropriate.

### Pre-implementation activities

Prior to implementation, we conducted informational sessions with facility leadership and Primary Care clinic administrators. We also developed four PowerPoint education files to educate the general neurologists about secondary stroke prevention evidence and updates from the 2021 American Heart/American Stroke Association Stroke Prevention Guidelines (22) Neurologists self-certified review of these educational materials. None of the general neurologists from NTNP who completed the stroke/TIA e-consults had additional vascular neurology certification.

### Stepped-wedge study design and randomization

**Figure 1:**
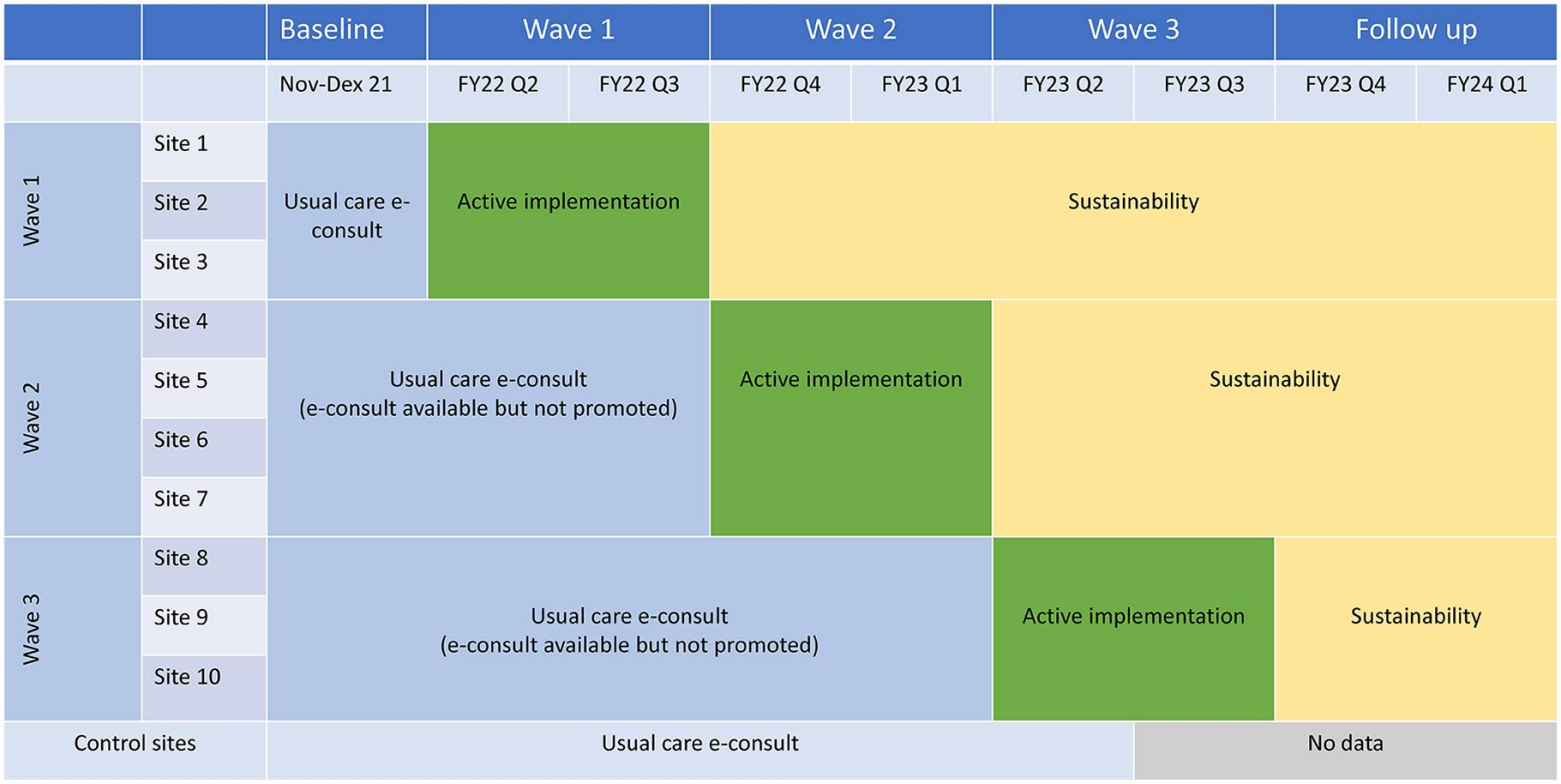
Stepped wedge study design.

This project was designed as a stepped-wedge cluster-randomized trial (Figure 1). We chose this design to evaluate the primary question of whether the implementation activities increased the proportion of outpatient stroke/TIA consults placed in the VA compared to CCN.

The e-consult was available at all NTNP sites at the same time (beginning of the baseline period). Those sites that were active in FY2021 or projected to go live in the NTNP program in early FY2022 with greater than 50 community-care stroke/TIA consults during FY2021 were randomized to receive the active implementation intervention. Sites were randomly assigned to active implementation waves using restricted randomization.

At the time of wave two, one of the randomized study sites that had projected to go live in early FY2022 had not yet started in the NTNP. Only one other new site (not active in NTNP at time of randomization) met study criteria and had since joined NTNP; this site was thus substituted in place of the randomized site to receive the intervention and was included in wave three. Any sites not active in NTNP at the time of wave 1 could be missing baseline data for some periods; this was accounted for in the power analysis.

Data from NTNP sites with less than 50 stroke/TIA CCN consults per year also had access to the e-consult at their facilities and were included in the project as “control sites” to assess the impact of any VA policy changes related to CCN consults during the study period.

### Implementation intervention

**Figure 2:**
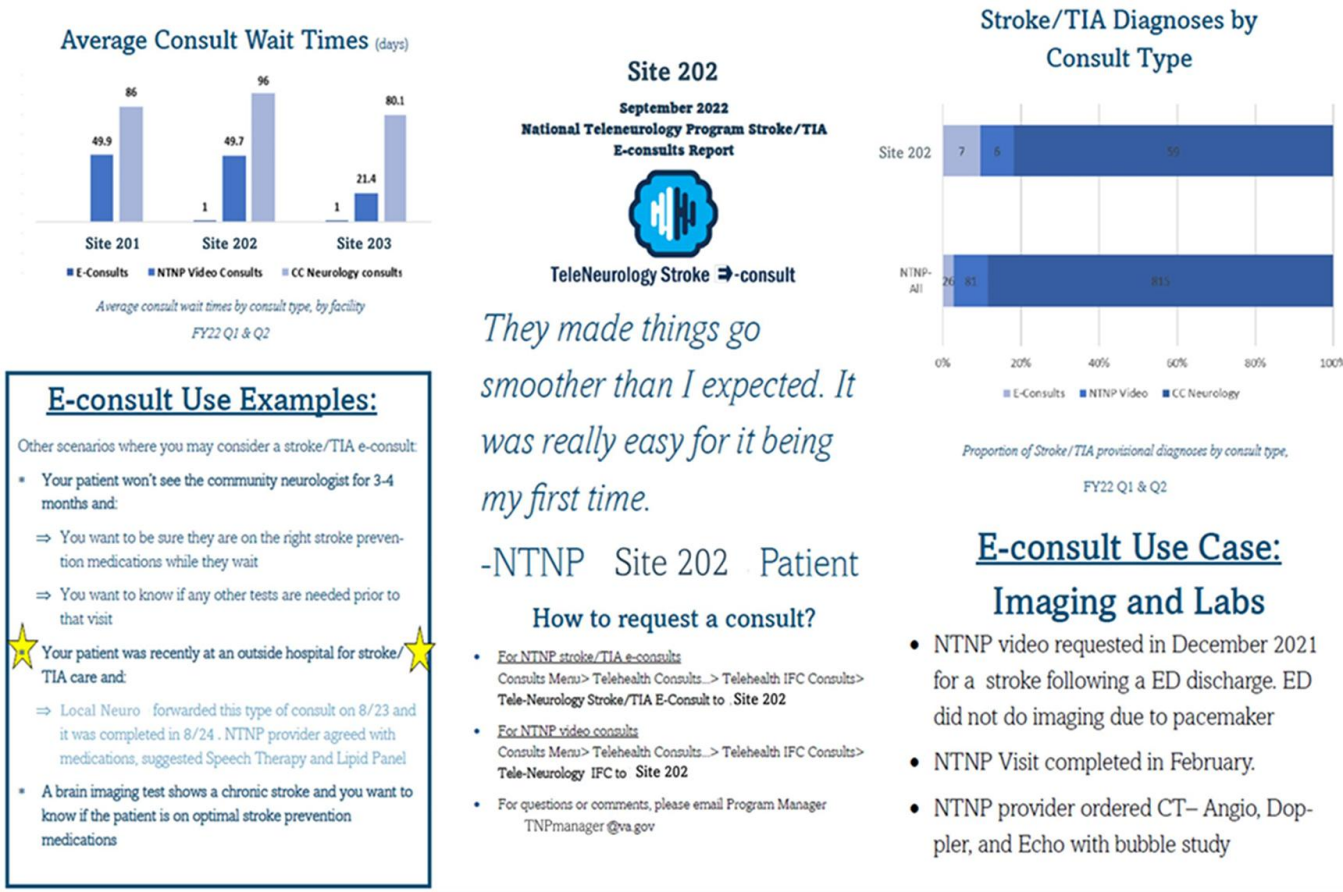
Example audit and feedback form.

Implementation strategies employed during the active implementation period were focused on PCP and clinic manager awareness of the e-consult, educating PCPs about common clinical scenarios where outpatient stroke/TIA e-consults could be useful, and PCP education about evidence-based stroke prevention strategies. We also held monthly audit and feedback sessions with behavioral nudges for PCPs and clinic managers that included: volume and wait times of NTNP e-consults and video consults for all sites active in that wave; volume and wait times of CCN consults for stroke care, and Veteran feedback about NTNP care (Figure 2) (22, 23).

We held meetings with facility and Primary Care clinical leadership one month prior to implementation to introduce the e-consult implementation plan and identify optimal methods of communication. During the implementation wave we attended monthly primary care team meetings where NTNP data sharing was part of the meeting agenda; our team reviewed these data with PCPs and clinic managers and provided monthly updated one-page data sheets in both digital form and in visual presentation during the meeting. We also offered to provide a one-hour didactic presentation to PCPs at each site during the active implementation wave; this presentation reviewed the American Heart Association 2021 Stroke Prevention Guidelines with a focus on updates related to new outpatient stroke prevention medication management strategies (24).

### Outcomes

The primary outcome was the proportion of all stroke/TIA consults (e-consult plus video consults) placed by PCPs for NTNP care with total stroke/TIA consults placed (NTNP plus CCN consults) as the denominator. This outcome did not account for whether the consult was completed. Stroke/TIA consults were identified based on the PCP's ICD10 diagnosis at the time the consult was placed.

Secondary outcomes of interest included stroke/TIA consults completed (e.g., the patient had a completed visit). Consult completion was considered a secondary outcome since other Veteran and health system variables may impact whether a consult is completed and thus diminish our ability to examine the impact of the implementation strategies on PCP decisions about the type of care to request.

### Analysis and study power

We used FY2020 data to estimate that 15 stroke/TIA consults would be placed per site during each two-month period over the 20 months of the study (*N* = 915). Power was calculated assuming two sites were not live during the initial baseline period (25, 26). We assumed that there would not be enough patients with multiple consults placed to account for correlation of observations from the same patient and thus only used the first consult during the observation period for any given patient in the analysis. Under these assumptions, and with the baseline estimate of the proportion of all stroke/TIA consults placed in the VA at 35%, power analysis with 80% power and type I error set at 0.05 determined a detectable difference in proportion of all consults placed in VA at 11.3% (46.3% of consults placed in VA). This difference ranged from 5.4% to 16.6% as the baseline estimate varied from 30% to 40%.

Due to the expected variation of sample size across the sites and across the implementation phases, we fit generalized linear mixed-effects models (GLMM) to the patient level data (27, 28). We used multilevel hierarchical random effects to analyze the intervention effects on the proportion of consults placed within the VA system during the post-implementation period and sustainability period compared with the baseline period. To examine the temporal trends in implementation effectiveness we also estimated the proportion of consults placed within the VA at each site for each two-month time period.

For the primary effectiveness analysis, the main effect of interest was the indicator for sites in the implementation phase vs. baseline phase. The effect of sustainability vs. baseline was also estimated although no formal power calculation was conducted for this comparison. Covariates considered potential confounders were selected *a priori* including: patient characteristics of age, co-morbidity as measured by the Charlson Comorbidity Index (CCI), race (white vs. non-white), and geographic region of residence (urban vs. non-urban) and site variables of in-person VA neurology available (yes vs. no), stroke/TIA volume at baseline, median CCN wait time at baseline (time from CCN consult placed to completed). A site-level indicator (intervention vs. control) was also included to account for any baseline difference between intervention and control sites. As “control” sites were an addition to the original model and only serve to aid in estimation of the secular time trend, the planned sensitivity analysis included re-running the final model excluding control sites.

As a secondary outcome, the proportion of consults for stroke/TIA diagnoses which were completed in the VA vs. community care (not just placed to VA or the community) was analyzed with a similar model as the primary outcome. The full results for this secondary outcome are provided in supplemental material; these models provided similar results to the primary outcome models. Maximum likelihood was used for parameter estimation and all data analyses were performed using SAS version 9.4 (SAS Institute, INC., Cary, NC, USA). A *p* value of < .05 was considered statistically significant.

## Results

A total of 1,680 consults were placed for stroke/TIA over the study period, of which 1,106 consults were placed during the baseline or active e-consult phase for intervention sites. Thus, this sample size is in alignment with our power analysis. Of the 1,680 consults, 22% (365/1,680) were placed to NTNP, lower than our baseline estimate of 35%. Among the VA consults, most were for video Teleneurology consult with only 61 (3.6%) submitted as e-consults (Table 1). Patients in the 10 intervention sites were on average 70.6 years old (SD 10.7); most were male (95.8%), white (88.7%), and a majority were rurally residing. Patient characteristics for the intervention sites were consistent across study periods. Patients from the control sites at baseline were slightly older (mean age, 71.4 years) and had slightly lower medical co-morbidity scores (median Charlson Comorbidity Index of 2, IQR 1–4). Among the intervention sites, half (five) had some limited local neurology presence (less than one full time neurologist) with FY2021 median wait time from date of neurology consult request to completion of 79.5 days (range 55–125 days).

**Table 1:** Summary of demographic characteristics and consults by study period.

	Study period			
	Baseline	Active	Sustainability	Overall
Patient demographics: Intervention Sites (*n* Consults = 1,545)				
Age (years): mean (SD)	70.6 (10.7)	70.5 (11.1)/ 73 (65,77)	71.2 (10.7)/ 73 (66,77)	70.7 (10.8)/ 73 (65,77)
CCI: mean (SD)/median (IQR)	3.2 (2.6)/3 (1,5)	3.0 (2.7)/2 (1,5)	2.9 (2.5)/2 (1,4)	3.0 (2.6)/3 (1,5)
Male, *n*(%)	549 (95.8%)	495 (93.8%)	423 (95.3%)	1,467 (95.0%)
White, *n*(%)	508 (88.7%)	478 (90.5%)	405 (91.2%)	1,391 (90.0%)
Urban, *n*(%)	239 (41.7%)	240 (45.5%)	197 (44.4%)	676 (43.8%)
Neurologist Stroke/TIA diagnosis, *n* (%)	252 (58.2%)	235 (61.7%)	140 (55.3%)	627 (58.8%)
Patient demographics: Control Sites (*n* Consults = 135)				
Age (years): mean (SD)/median (IQR)	71.4 (10.1)/ 72 (66,76)	–	–	–
CCI: mean (SD)/median (IQR)	2.7 (2.2)/2 (1,4)	–	–	–
Male, *n* (%)	128 (94.8%)	–	–	–
White, *n* (%)	116 (85.9%)	–	–	–
Urban, *n* (%)	70 (51.9%)	–	–	–
Neurologist Stroke/TIA diagnosis, *n* (%)	63 (66.3%)	–	–	–
Consult Volume				
	*n* = 708	*n* = 528	*n* = 444	*n* = 1,680
NTNP E-consults placed	14 (2.0%)	29 (5.5%)	18 (4.0%)	61 (3.6%)
NTNP video consults placed	137 (19.3%)	97 (18.4%)	70 (15.8%)	304 (18.1%)
CCN consults placed	557 (78.7%)	402 (76.1%)	356 (80.2%)	1,315 (78.3)

Based on the GLM model with random effects fit to the patient-level data on consults placed, and including active plus control sites, the odds of a stroke/TIA consult being placed within the VA during active implementation increased 48% (OR = 1.48, 95% CI, 0.96–2.28, *p* = 0.074) compared to the baseline period (Table 2). In the sensitivity analysis without control sites added, the odds of the consult being placed in VA remained and was statistically significantly higher during active implementation (OR = 1.92, 95% CI 1.17–3.16, *p* = 0.010). Of the covariates only age was statistically significant, with older patients less likely to have their consult placed within the VA (OR = 0.99, 95% CI, 0.98–1.00, *p* = 0.024). There was no significant increase in VA consults in the sustainability period compared to baseline in either model.

**Table 2:** Model of consults placed.

	All sites (*n* = 1,680)		Intervention sites only (*n* = 1,545)	
Effect	Odds Ratio Estimates (95% CI)	Pr > |t|	Odds Ratio Estimates (95% CI)	Pr > |t|
Assignment: Intervention vs Control	0.38 (0.13–1.11)	0.076	–	–
Phase: Active-implementation vs Baseline	1.48 (0.96–2.28)	0.074	**1.92** (**1.17–3.16)**	**0**.**010**
Phase: Sustainability vs. Baseline	1.22 (0.63–2.36)	0.547	1.85 (0.83–4.13)	0.134
Age	0.99 (0.98–1.00)	0.024	0.99 (0.98–1.00)	0.028
CCI	0.99 (0.95–1.05)	0.845	1.00 (0.95–1.05)	0.963
Race: White vs. Nonwhite	1.03 (0.69–1.55)	0.884	0.94 (0.61–1.44)	0.766
Urban: Yes vs. No	0.98 (0.76–1.26)	0.866	0.94 (0.72–1.23)	0.647
LocalNeurology: Yes vs. No	0.84 (0.39–1.81)	0.662	0.98 (0.36–2.63)	0.961
Stroke TIA volume	1.01 (0.99–1.02)	0.508	1.01 (0.99–1.02)	0.492
Median Wait Time	1.00 (0.98–1.01)	0.643	1.00 (0.97–1.02)	0.671
TimeBlock: 2 vs. 1	0.62 (0.35–1.09)	0.099	0.41 (0.22–0.78)	0.006
TimeBlock: 3 vs. 1	0.42 (0.22–0.77)	0.005	0.29 (0.15–0.59)	<.001
TimeBlock: 4 vs. 1	0.81 (0.46–1.43)	0.462	0.56 (0.30–1.04)	0.065
TimeBlock: 5 vs. 1	0.58 (0.31–1.10)	0.097	0.36 (0.17–0.75)	0.007
TimeBlock: 6 vs. 1	0.77 (0.41–1.46)	0.421	0.51 (0.24–1.08)	0.080
TimeBlock: 7 vs. 1	1.06 (0.57–1.96)	0.859	0.71 (0.34–1.46)	0.347
TimeBlock: 8 vs. 1	0.72 (0.35–1.50)	0.380	0.40 (0.16–0.98)	0.044
TimeBlock: 9 vs. 1	0.71 (0.35–1.47)	0.356	0.35 (0.14–0.87)	0.024
TimeBlock: 10 vs. 1	0.62 (0.30–1.26)	0.184	0.34 (0.14–0.82)	0.016

Bold values indicate significant results in the main contrast of interest.

For the secondary outcome of proportion of consults completed within VA (shown in Supplementary Table S1), 1,257 consults were completed of which 265 (21%) were NTNP within the VA. For this outcome, the effect of the active e-consultation on consults completed was similar to the primary outcome results, with the odds of a consult being completed within the VA more likely during active implementation (OR = 1.57, 95% CI, 0.96–2.58, *p* = 0.074), and with a statistically significant increase in likelihood of VA consult during active implementation in the sensitivity analysis (OR = 1.93, 95% CI, 1.08–3.45, *p* = 0.026).

## Discussion

This quality improvement evaluation demonstrates that the implementation of an e-consult for outpatient stroke/TIA care can increase the proportion of neurology consults for stroke/TIA placed to VA compared to consults placed for community care. The effect estimate was an increase of at least 50% during the active implementation period, but this increase was not statistically significant once active implementation ended.

Our active implementation strategies included provider and administrator education and monthly audit and feedback where the implementation team joined regularly scheduled PCP meetings to provide short updates on consult usage and wait times, as well as Veteran feedback about NTNP care. Although we cannot disentangle specific effects of different parts of this intervention, we know that audit and feedback of clinic data can lead to small changes in care processes, especially when feedback is timely, individualized (provider and/or clinic-specific), and non-punitive (29, 30). Our data feedback did include some features associated with effective audit and feedback in a recent Cochrane review (30), including data on a high priority clinical issues (wait times), including written and verbal feedback, comparison to peers (data compared to CCN and to other VA sites), and the inclusion of an action reminder (how to place an NTNP e-consult). It may be that the regular presentation of these data, even though lasting typically only a few minutes in a monthly PCP team meeting, served as a reminder of NTNP care options and led to an increase in PCP recommendations to the Veteran for NTNP care.

The increase in NTNP consults placed during active implementation was largely due to increased consults for standard video visits rather than large numbers of new e-consults being placed (there were only 29 e-consults during active implementation and 18 during sustainability). A possible reason for the increased NTNP video consults during active implementation is the repeated presentation of NTNP vs. CCN wait times in the audit and feedback tool. Overall data on wait times during this period showed that, although e-consults were completed rapidly (median of three days), NTNP video consult completion times were also significantly faster than CCN consult completion (median 44 days for NTNP video consults compared to 97 days for CCN consults in FY20–21) (13); perhaps leading providers to place a video consult instead of the even quicker e-consult. It is also possible that the monthly presentation of data to clinic administrators, including schedulers who directly assist Veterans with choosing from available care options, may have increased the likelihood that the wait time difference was clearly communicated to Veterans eligible for non-VA care, thus influencing their choice.

Considerations around potential benefits of continuity of care within a single system may also influence where providers choose to place a consult and where Veterans choose to receive their care. Although we did not directly ask about factors related to choice of care in this project, prior qualitative studies about the impacts of expanding Veteran care to the community found both patients and providers reported concerns about reduced coordination of care when Veterans are referred for care in the community, including difficulty sharing test results and obtaining outside prescriptions, and physician burnout due to difficulty accessing community records (18–20). Our analysis focused on where consults were placed, and we do not have qualitative data from providers to know what factors shaped their decision about placing an NTNP e-consult vs. NTNP video consults or CCN consults.

Finally, our findings are similar to those reported in a stepped-wedge cluster randomized study of specialty e-consult implementation in a non-VA healthcare system. This intervention, which included incentive payments to PCPs, led to widespread adoption of e-consults but demonstrated no corresponding decrease in “regular” specialty care referrals (31). This suggests that the addition of an e-consult option alone may not be adequate to significantly shift existing consultation practices away from other available referral options. Telehealth care, including both video visits and e-consults, have been common in the VHA for over a decade, with growth in telehealth, including e-consultations, expanding rapidly during the COVID-19 pandemic (32). Specialty care e-consultation is also common in VA, with positive impacts on access, but noted concerns among VA primary care providers about the additional burden imposed by this method of consultation (33, 34). The degree to which provider workload concerns may have impacted the number of e-consults vs. “usual” video Teleneurology consults for stroke/TIA care in this project is not known.

VA telehealth has fewer barriers to implementation than telehealth care in non-VA systems, which could make generalizing our findings outside the VA somewhat challenging. However, our observation about the effect of pragmatic efforts to bolster audit and feedback of telehealth data on choices about where consults are placed may have relevance for non-VA healthcare systems. If, for example, large urban academic healthcare systems promote a stroke/TIA e-consult to community PCPs after their patient's inpatient stroke admission, there could be a similar effect on increasing outpatient neurology consultation within that healthcare system and promoting improved care coordination and transitions of care post-stroke. This may be increasingly important in settings where patients have multiple choices about the location of their post-hospital follow-up. Another limitation of our project is that we also included “control” sites that were not part of the stepped wedge trial. We included these sites to ensure we could assess the effect of any possible national changes in eligibility criteria or other processes for obtaining non-VA care during the study period. However, no temporal changes were noted and the variability in these smaller control sites resulted in the implementation effect slightly exceeding the significance criteria set in our model that included all NTNP sites. Our study did not find that the impact of the intervention was sustained, although the post-implementation time period varied by wave and we were not powered to examine sustainability during a shorter time frame when it might be more likely that intervention effects could potentially remain. Future implementation efforts around e-consults could consider the addition of pragmatic sustainment strategies that may continue to bolster use of the available telehealth consultation modalities and could assess sustainability over both short- and longer post-implementation periods.

Despite these limitations, we conclude that the implementation of a new e-consult for outpatient stroke/TIA care increased the proportion of neurology consults placed within VA care compared to care in the community during the period of active implementation. Sustaining this effect and increasing the total number of e-consultations placed may require additional resources for ongoing implementation activities and will require a deeper understanding of the factors that underlie both patient and provider choices about the desired location of care. Given what we know about the quality of VA care, Veteran care preferences, and the benefits of care coordination within an integrated healthcare system, interventions to increase Veterans’ access to scarce specialty care within the VA should continue to be implemented and evaluated. Subsequent planned analyses from this project will compare the quality of outpatient stroke/TIA care and Veteran satisfaction with care in these different care settings, outcomes that are critically important to Veterans and their referring providers.

## Data Availability

The datasets presented in this article are not readily available because data are the property of the Department of Veterans Affairs. Requests to access the datasets should be directed to Linda.Williams6@va.gov.
