# Supplementary material for: Implementation of a stroke/TIA e-consult increases outpatient stroke Teleneurology consults placed in VHA compared to community care
Source: Front Health Serv. 2026 Jul 22;6:1839089. doi: 10.3389/frhs.2026.1839089 (PMC13437968; doi:10.3389/frhs.2026.1839089)
Supplement: Supplementary file 1 [file Datasheet1.pdf]

Supplementary Materials. “Implementation of a Stroke/TIA e-consult Increases Outpatient Stroke Teleneurology Consults placed in VHA compared to Community Care”

Supplemental Table 1. Results for consults completed from GLMM

	all the sites (n=1257)		intervention sites only (n=1151)	
Effect	Odds Ratio Estimates (95% CI)	Pr > |t|	Odds Ratio Estimates (95% CI)	Pr > |t|
Assignment: Intervention vs Control	0.36 (0.12 - 1.11)	0.076	---	---
**Phase: Active-implementation vs Baseline**	1.57 (0.96 - 2.58)	0.074	**1.93 (1.08 - 3.45)**	**0.026**
Phase: Sustainability vs. Baseline	1.29 (0.61 - 2.74)	0.507	1.76 (0.69 - 4.53)	0.239
Age	0.99 (0.98 - 1.00)	0.119	0.99 (0.98 - 1.00)	0.167
CCI	0.98 (0.92 - 1.04)	0.486	0.98 (0.91 - 1.04)	0.500
Race: White vs. Nonwhite	1.05 (0.64 - 1.74)	0.835	0.99 (0.58 - 1.69)	0.965
Urban: Yes vs. No	0.85 (0.63 - 1.16)	0.309	0.87 (0.63 - 1.19)	0.380
Local Neurology: Yes vs. No	0.80 (0.36 - 1.74)	0.569	0.85 (0.30 - 2.37)	0.750
Stroke TIA volume	1.00 (0.99 - 1.02)	0.617	1.00 (0.99 - 1.02)	0.698
Median Wait Time	0.99 (0.98 - 1.01)	0.502	0.99 (0.97 - 1.02)	0.569
TimeBlock: 2 vs. 1	0.69 (0.35 - 1.35)	0.278	0.46 (0.22 - 0.99)	0.047
TimeBlock: 3 vs. 1	0.39 (0.19 - 0.82)	0.013	0.29 (0.13 - 0.67)	0.004
TimeBlock: 4 vs. 1	0.83 (0.42 - 1.64)	0.589	0.69 (0.33 - 1.44)	0.323
TimeBlock: 5 vs. 1	0.66 (0.32 - 1.36)	0.256	0.44 (0.18 - 1.03)	0.059
TimeBlock: 6 vs. 1	0.85 (0.40 - 1.81)	0.672	0.64 (0.26 - 1.59)	0.340
TimeBlock: 7 vs. 1	1.04 (0.50 - 2.13)	0.920	0.82 (0.35 - 1.90)	0.638
TimeBlock: 8 vs. 1	0.77 (0.33 - 1.80)	0.543	0.52 (0.18 - 1.51)	0.231
TimeBlock: 9 vs. 1	0.90 (0.39 - 2.06)	0.799	0.49 (0.17 - 1.44)	0.194
TimeBlock: 10 vs. 1	0.75 (0.33 - 1.71)	0.497	0.48 (0.17 - 1.36)	0.167
